# Climate risk or healthcare investment? Explaining oral health outcomes across European Union member states

**DOI:** 10.3389/fpubh.2026.1939057

**Published:** 2026-09-30

**Authors:** Cassandra Lupita, Anca-Cristina Perpelea, Laura-Cristina Rusu, Iulia Muntean, Edida Maghet, Oana Ramona Lobonț, Magda-Mihaela Luca

**Affiliations:** 1 Department of Oral Pathology, Multidisciplinary Center for Research, Evaluation, Diagnosis and Therapies in Oral Medicine, “Victor Babes” University of Medicine and Pharmacy Timisoara, Timisoara, Romania; 2 Department of Organization, Professional Legislation and Management of the Dental Office, Faculty of Dental Medicine, “Carol Davila” University of Medicine and Pharmacy, Bucharest, Romania; 3 Finance, Business Information Systems and Modeling Department, Faculty of Economics and Business Administration, West University of Timisoara, Timisoara, Romania; 4 Department of Pediatric Dentistry, Faculty of Dental Medicine, “Victor Babes”, University of Medicine and Pharmacy Timisoara, Timisoara, Romania

**Keywords:** climate change, dental caries, extreme heat days, general government health expenditure, oral health, panel data analysis, periodontal disease, unmet dental care needs

## Abstract

**Introduction:**

Climate change is a major public-health concern, but short-term population-level evidence linking annual climate variability to oral-health outcomes remains limited. This study evaluates within-country associations between temperature, extreme heat days, and three oral-health indicators across EU member states.

**Methods:**

A longitudinal ecological panel analysis was conducted for the 27 EU member states from 2016 to 2023. Daily temperatures at each country centroid were obtained from the Open-Meteo Historical Weather API and aggregated to annual mean temperature and days with maximum temperature above 30 °C. Outcomes were GBD 2023 carries and periodontal prevalence and Eurostat unmet dental care. Two-way fixed-effects models used country-clustered finite-sample-corrected standard errors and 999-replication wild-cluster bootstrap *p*-values.

**Results:**

Neither mean annual temperature nor extreme heat days were associated with any outcome in the two-way fixed-effects models. Within R-squared values were 0.056 for caries, 0.032 for periodontal disease, and 0.044 for unmet dental care. Wild-cluster bootstrap inference confirmed the absence of associations at the 5% level. Quadratic temperature terms and climate-by-expenditure interactions were also non-significant.

**Conclusion:**

Over the eight-year study period, short-term within-country variation in the reconstructed climate indicators was not associated with the examined oral-health outcomes. Nonlinear and expenditure-interaction models were also null. Longer panels, spatially resolved exposure measures, and oral-health-specific financing, coverage, and utilization indicators are needed.

## 1 Introduction

Climate change is one of the most serious challenges impacting global public health and is associated with documented effects on multiple health outcomes, including cardiovascular diseases, respiratory disorders, and infectious illnesses (1–3). Rising average global temperatures and increasing instances of heat waves (numbers of extreme heat days) have been linked to an array of physiological and systemic consequences, either directly or indirectly (4, 5). Recently, attention has been drawn toward the identification of health domains where associations with environmental exposures, especially with respect to climate changes, are understudied.

One area where there is an evident lack of knowledge about environmental effects is that of oral health. While a significant amount of literature has been published regarding socioeconomic, behavioral, and health care determinants of oral diseases, the possible impact of environmental and climatic variables has received much less consideration from the population health standpoint (6, 7). This issue becomes especially relevant because oral diseases are some of the most prevalent non-communicable diseases and pose a serious burden on health care services (6, 8).

Biologically, there are several factors that could potentially connect the exposure to climate stressors and oral health outcomes. First, exposure to high temperature and extreme heat days could result in dehydration and disruption of electrolytes balance, which would affect saliva production and its functioning (9–11). Saliva has an important role in maintaining oral homeostasis because of its buffering, antibacterial effects, and ability to remineralize (12, 13). Changes in both quality and quantity of saliva would likely result in increased risk for various oral diseases such as caries and periodontal diseases (12–14). Moreover, heat stress might influence behavior and utilization of oral health services (15).

In addition to biological processes, research shows that effects of climate stressors on oral health can be influenced by institutional structures as well, since prevention, diagnostics and treatment of oral diseases require significant investments in the development of the public health infrastructure and dental care provision (16–18). Hence, the impacts of climate exposure on oral health are expected to vary widely across countries.

The European Union, with its diverse climate and healthcare systems, can be considered an appropriate environment for such analysis. However, there is little existing literature combining climate exposure, oral health outcomes, and structural determinants within a unified panel dataset (19, 20).

Accordingly, this study examined whether within-country changes in mean annual temperature and extreme heat days were associated with changes in untreated caries of permanent teeth, periodontal diseases, and unmet dental-care needs across the EU-27 during 2016–2023. General government health expenditure as a percentage of GDP was included as a time-varying structural covariate. The primary objective was to distinguish pooled geographical patterns from longitudinal associations identified from changes within countries; the study was not designed to establish individual-level mechanisms, causal effects, or a climate-mitigating role of healthcare expenditure.

By applying a within-country panel framework, the study contributes population-level evidence on an under-investigated topic while explicitly recognizing the limits of national aggregate data and the short observation period.

## 2 Materials and methods

### 2.1 Study design

The current study uses a longitudinal ecological panel design for examining the relationship between climate-related stressors and oral health outcomes among European Union member countries. The country serves as the unit of analysis, where each country is assessed yearly to form a panel dataset that captures both cross-sectional and temporal variation. The ecological approach is suitable for studying population-level trends because of the possibility to study the impact of structural and environmental elements of a country, including climate conditions and health care systems.

The use of a panel dataset makes it possible to use fixed-effects models to account for unobserved time-invariant characteristics specific to countries and can help identify temporal dynamics in countries. Nonetheless, being an ecological study, the present study is limited by the issues associated with aggregation; thus, only population-level associations rather than individual-level causal effects will be discussed. This design is particularly appropriate for assessing structural and environmental determinants that vary across countries and over time.

### 2.2 Study period and data structure

This paper analyzes the data from 2016 to 2023 and focuses on all 27 European Union member countries. The data consists of a nearly balanced panel of country-year observations, with a maximum of 216 observations depending on data availability across variables.

Each observation corresponds to country i in year t, forming a two-dimensional panel structure that captures both cross-sectional differences between countries and temporal changes within countries. All data is measured at the country level and harmonized annually for consistency and comparability between observations within and across countries.

The selected study period was chosen to ensure consistent data availability across climate and health indicators and to capture recent trends in climate variability and population health outcomes within the European Union.

### 2.3 Data sources

Data from internationally acknowledged and harmonized datasets were collected to ensure consistency and comparability between countries.

Daily temperature observations were obtained from the Open-Meteo Historical Weather API (21) for the geographic centroid coordinates of each EU member state. Daily mean temperature was averaged within each country-year, and extreme heat days were counted as days with maximum temperature above 30 °C. This reproducible extraction produced 216 country-year observations. The centroid-based exposure is a national climatic proxy and does not capture within-country spatial heterogeneity.

Oral-health estimates were obtained from the Institute for Health Metrics and Evaluation (IHME) Global Burden of Disease 2023 results (23). Both-sex prevalence series were used: caries of permanent teeth for all ages and periodontal diseases using age-standardized estimates. Values downloaded as proportions were multiplied by 100 and analyzed as percentage points. Unmet dental care was obtained from Eurostat EU-SILC (hlth_silc_09) as the percentage of people aged 16 years or older reporting unmet dental examination needs due to cost, distance, or waiting lists. General government health expenditure as a percentage of GDP was obtained from the World Bank indicator SH.XPD.GHED.GD.ZS (24).

Variables were harmonized to an annual frequency. Data processing included verification of data, country code harmonization, and temporal alignment to create a harmonized panel database for econometric analysis (23–25).

### 2.4 Variables

The variables included in the analysis, along with their definitions, units of measurement, data sources, and role in the model, are presented in Table 1.

**Table 1:** Definition of variables, units of measurement, data sources, and role in the model.

Variable	Definition	Unit	Source	Role
Mean annual temperature	Annual average air temperature	°C	Copernicus Climate Data Store (ERA5)	Independent
Extreme heat days	Number of days with maximum temperature exceeding 35 °C	days/year	Copernicus Climate Data Store (ERA5)	Independent
Untreated dental caries	Model-based prevalence estimate of untreated dental caries in the population	%	Global Burden of Disease (IHME)	Dependent
Periodontal disease	Model-based prevalence estimate of periodontal disease in the population	%	Global Burden of Disease (IHME)	Dependent
Unmet dental care needs	Share of population reporting unmet dental care needs	%	Eurostat (EU-SILC)	Dependent
General government health expenditure	General government health expenditure	% of GDP	Eurostat	Control

The main independent variables mean annual temperature (°C) and extreme heat days (days/year). The dependent variables included untreated dental caries prevalence (%), periodontal disease prevalence (%), and unmet dental care needs (%). All outcome variables were expressed as annual prevalence estimates at the national level.

General government health expenditure (% of GDP) was included as a broad time-varying indicator of health-system financing. It does not measure dental expenditure, preventive-care expenditure, service coverage, or healthcare-system capacity directly, and changes may reflect variation in either expenditure or GDP.

All oral-health outcomes were analyzed and reported on the 0–100 percentage-point scale. Mean annual temperature was expressed in degrees C, extreme heat in days per year, and general government health expenditure as percentage of GDP. No logarithmic transformation or standardization was applied in the primary models.

Although xerostomia represents a biologically plausible intermediate mechanism linking climate stressors to oral health outcomes, it was not included in the econometric model due to the lack of comparable data at the EU level. Instead, it is addressed within the conceptual framework and discussed as a potential mediating factor.

This limitation reflects the ecological nature of the study, where individual-level biological indicators were not available for consistent cross-country comparison.

### 2.5 Statistical model

The relationship between climate stressors and oral health outcomes was estimated using a fixed-effects panel data model with country and year effects, specified as follows Equation 1:


$$\begin{array}{l} Y_{\mathrm{it}}=\alpha +\beta_{1}{\text{Temperature}}_{\mathrm{it}}+\beta_{2}{\text{HeatDay}}_{\mathrm{it}}\\ +\gamma {\text{HealthExpenditure}}_{\mathrm{it}}+\mu_{i}+\lambda_{t}+\varepsilon_{\mathrm{it}} \end{array}$$ (1)

where *Y_it_* represents the oral health outcome in country *_i_* at time *_t_*, Temperature_it_ is the mean annual temperature, *HeatDays_it_* is the number of extreme heat days, and *HealthEpenditure_it_* represents general government health expenditure as a percentage of GDP, *μ_i_* captures unobserved country-specific effects, *λ_t_* represents time-specific effects, and *ε_it_* is the error term.

Separate fixed-effects models were estimated for each dependent variable, including untreated dental caries prevalence, periodontal disease prevalence, and unmet dental care needs.

Two-way fixed effects were selected *a priori* because the research question concerned changes within countries and because persistent country characteristics, including geography, institutional structure, diet, and healthcare organization, may be correlated with the included covariates. Country effects control for time-invariant differences and year effects control for common temporal shocks. Standard errors were clustered by country. With only 27 clusters and eight annual observations per country, statistical inference remains imprecise and *p*-values should be interpreted cautiously.

### 2.6 Statistical tests and diagnostics

In order to verify the validity of the estimated panel models, several diagnostic tests and specification checks have been carried out. All statistical analyses were conducted using Python (version 3.14), primarily relying on the pandas, stats models, and linear models’ libraries for data management, descriptive statistics, and panel regression analysis.

The fixed-effects specification was selected *a priori* because the concerns within-country change and persistent country characteristics may correlate with the covariates. We report the within R-squared for each model. Country-clustered covariance estimates used the finite-sample correction G/(G − 1) × (N − 1)/(N − K), with inference based on t (22). In addition, 999-replication Rademacher wild-cluster bootstrap *p*-values were calculated for the three focal coefficients.

Residual diagnostics included an AR (1) residual regression as a check for serial dependence and the Pesaran CD statistic for contemporaneous cross-country dependence. Because serial dependence was detected, inference emphasizes country-clustered and wild-cluster bootstrap results. The periodontal model also showed cross-sectional dependence; this is retained as a limitation rather than treated as resolved by clustering.

Within-country standard deviations were calculated to document identifying variation. Sensitivity analyses added a squared temperature term and, separately, temperature-by-expenditure and heat-day-by-expenditure interactions. These prespecified checks address possible nonlinearity and whether expenditure modifies the association between climate exposure and oral-health outcomes.

The AR (1) residual coefficients were 0.649 for caries, 0.737 for periodontal disease, and 0.422 for unmet dental care (all *p* < 0.001). Pesaran CD tests indicated cross-sectional dependence for periodontal disease (CD = 10.07, *p* < 0.001), but not clearly for caries (CD = −1.95, *p* = 0.052) or unmet dental care (CD = −0.37, *p* = 0.710). These diagnostics reinforce the exploratory interpretation of inference in this short macro-panel.

The within R-squared values were 0.056 for caries, 0.032 for periodontal disease, and 0.044 for unmet dental care. These values quantify the share of two-way-demeaned outcome variation explained by the time-varying covariates and are distinct from overall fit dominated by country effects.

Accordingly, the results are interpreted as exploratory longitudinal associations. The short temporal dimension, limited number of country clusters, and potential residual dependence restrict statistical power and precision.

### 2.7 Sensitivity analyses

To assess the robustness of the estimated relationships, a series of sensitivity analyses were conducted using alternative model specifications and variable configurations.

First, quadratic temperature models were estimated to assess nonlinearity. The squared temperature terms were non-significant for caries (*p* = 0.199), periodontal disease (*p* = 0.758), and unmet dental care (*p* = 0.315).

Second, effect-modification models included temperature-by-expenditure and extreme-heat-days-by-expenditure interactions. Neither interaction was significant at the 5% level for any outcome; the smallest *p*-value was 0.099 for the temperature-by-expenditure term in the caries model.

Third, wild-cluster bootstrap *p*-values for temperature, extreme heat days, and expenditure were, respectively, 0.127, 0.293, and 0.079 for caries; 0.113, 0.783, and 0.131 for periodontal disease; and 0.546, 0.805, and 0.125 for unmet dental care. Thus, none of the primary associations met the 5% threshold under small-cluster-robust inference.

Taken together, the sensitivity analyses did not identify robust nonlinear or effect-modification patterns. Given the number of outcomes and predictors, all nominal *p*-values are interpreted as exploratory.

### 2.8 Ethical considerations

This study is based exclusively on aggregated, publicly available data obtained from international databases and does not involve identifiable human subjects. As a result, ethical approval was not required in accordance with standard research guidelines for secondary data analysis and institutional policies governing research using anonymized datasets.

All data sources used in the study are anonymized and comply with relevant data protection and privacy regulations. No individual-level information was accessed or processed at any stage of the analysis.

From a methodological perspective, the ecological design introduces inherent limitations. Specifically, the use of aggregated data may lead to ecological fallacy, whereby relationships observed at the population level do not necessarily reflect individual-level mechanisms. Therefore, the findings should be interpreted as population-level associations rather than evidence of causal relationships at the individual level.

## 3 Results

### 3.1 Descriptive statistics

Descriptive statistics of all variables used in the empirical analyses are reported in Table 2. The panel data consist of 27 European Union (EU) member states over the time frame 2016–2023, which leads to 216 country-year observations on most variables.

**Table 2:** Descriptive statistics of the variables included in the panel dataset for EU-27 countries over the period 2016–2023.

Variable	Mean	Std. Dev.	Min	Max	*N*
Mean annual temperature (°C)	10.792	3.917	4.244	21.085	216
Extreme heat days (days/year)	19.782	34.677	0.000	160.000	216
Untreated caries prevalence (%)	37.480	6.870	22.077	47.658	216
Periodontal disease prevalence (%)	11.199	4.020	2.403	22.975	216
Unmet dental care needs (%)	3.502	3.216	0.100	14.200	215
General government health expenditure (% of GDP)	6.263	1.697	2.746	10.122	216

Mean annual temperature averaged 10.792 °C (SD 3.917; range 4.244–21.085), while extreme heat days averaged 19.782 per year (SD 34.677; range 0–160). Their within-country standard deviations were much smaller—0.513 °C and 6.253 days—illustrating that identification in the fixed-effects models relies on limited temporal variation.

Untreated caries prevalence averaged 37.480% (SD 6.870; range 22.077–47.658), age-standardized periodontal disease prevalence averaged 11.199% (SD 4.020; range 2.403–22.975), and unmet dental care averaged 3.502% (SD 3.216; range 0.100–14.200). The corresponding within-country standard deviations were 0.302, 0.249, and 1.178 percentage points.

General government health expenditure averaged 6.263% of GDP (SD 1.697; range 2.746–10.122), with a within-country standard deviation of 0.565 percentage points.

Overall, the relatively high standard deviations observed for most variables indicate substantial heterogeneity in the dataset, supporting the use of a panel data framework to capture both cross-sectional and temporal variation.

### 3.2 Climate trends in European Union member states

The trends of climatic indicators in the EU member states between 2016 and 2023 demonstrate various features that are reflected by changes in time and diversity in different countries.

The average annual temperature varies greatly in space since it is characterized by cold winters in the northern regions and hot summers in the southern areas of Europe. In terms of time, a positive correlation was observed in this case, and the average temperature of EU-27 increased from 11.2 °C in 2016 to 12.0 °C in 2023 (Figure 1).

**Figure 1:**
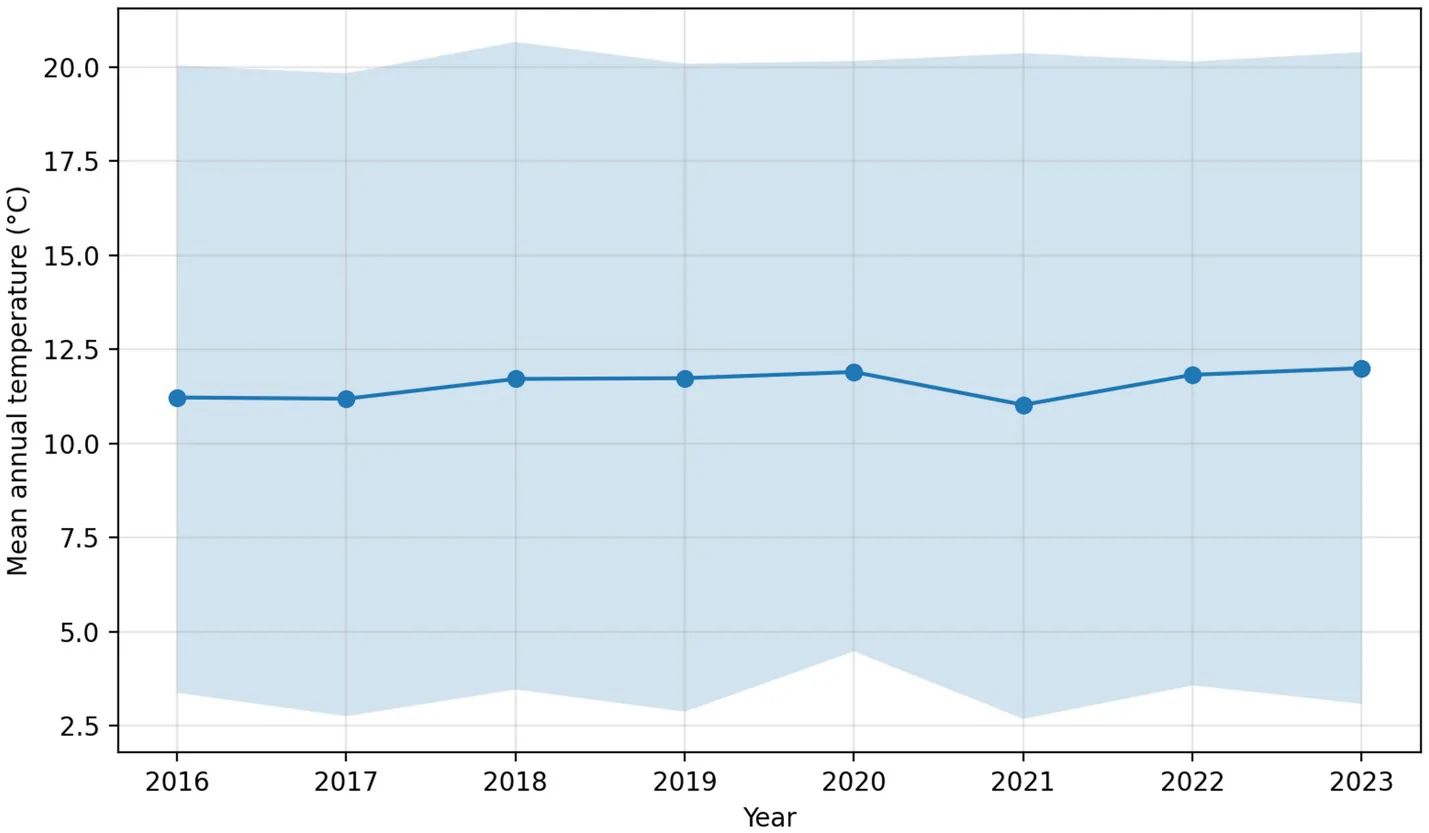
Evolution of mean annual temperature across European Union (EU-27) member states during the period 2016–2023. The solid line represents the EU-27 average, while the shaded area indicates the minimum–maximum range across countries.

Extreme heat days differ in greater fluctuations and demonstrate an increase compared to temperature anomalies (refer to Figure 2). While previous years were characterized by low figures, new year’s, in particular, 2022, were marked by high indicators which on average equaled 21 days a year for EU-27 countries.

**Figure 2:**
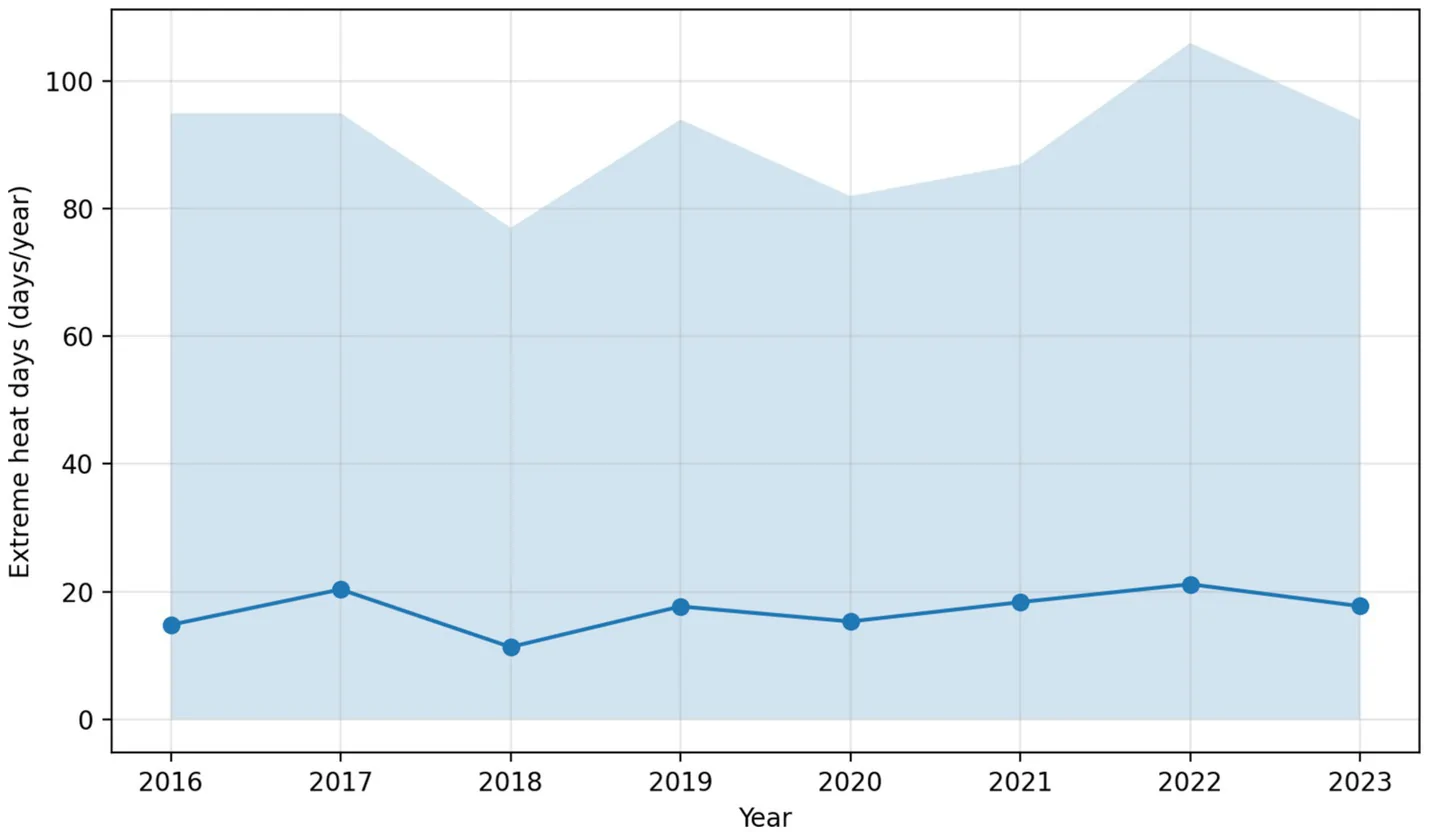
Evolution of extreme heat days across European Union (EU-27) member states during the period 2016–2023. The solid line represents the EU-27 average, while the shaded area indicates the minimum–maximum range across countries.

Member countries exhibit much variation in this case. For example, countries located to the south and south-east such as Italy, Greece, and Spain experience hotter temperatures and have higher cases of heat days than other member countries. However, in contrast, countries that belong to the north such as Sweden and Finland have low values for the two variables.

In general, the above analysis indicates an increasing trend in pressure because of climate changes characterized by temperature increases and heat days. Variation is very useful in analyzing the relationship between climate change and oral diseases.

### 3.3 Oral health trends in European Union member states

There exist quite significant differences in the level of oral health indicators development in different member countries during the period from 2016 to 2023. The percentage of untreated carious teeth is rather high for all the analyzed member countries and can range approximately between 22.1 and 47.7%, with its average value being approximately equal to 37.5%. No significant trends can be noticed regarding time as the indicator has no tendency to increase or decrease. Instead, there is some level of fluctuation, making the indicator rather stable (Figure 3).

**Figure 3:**
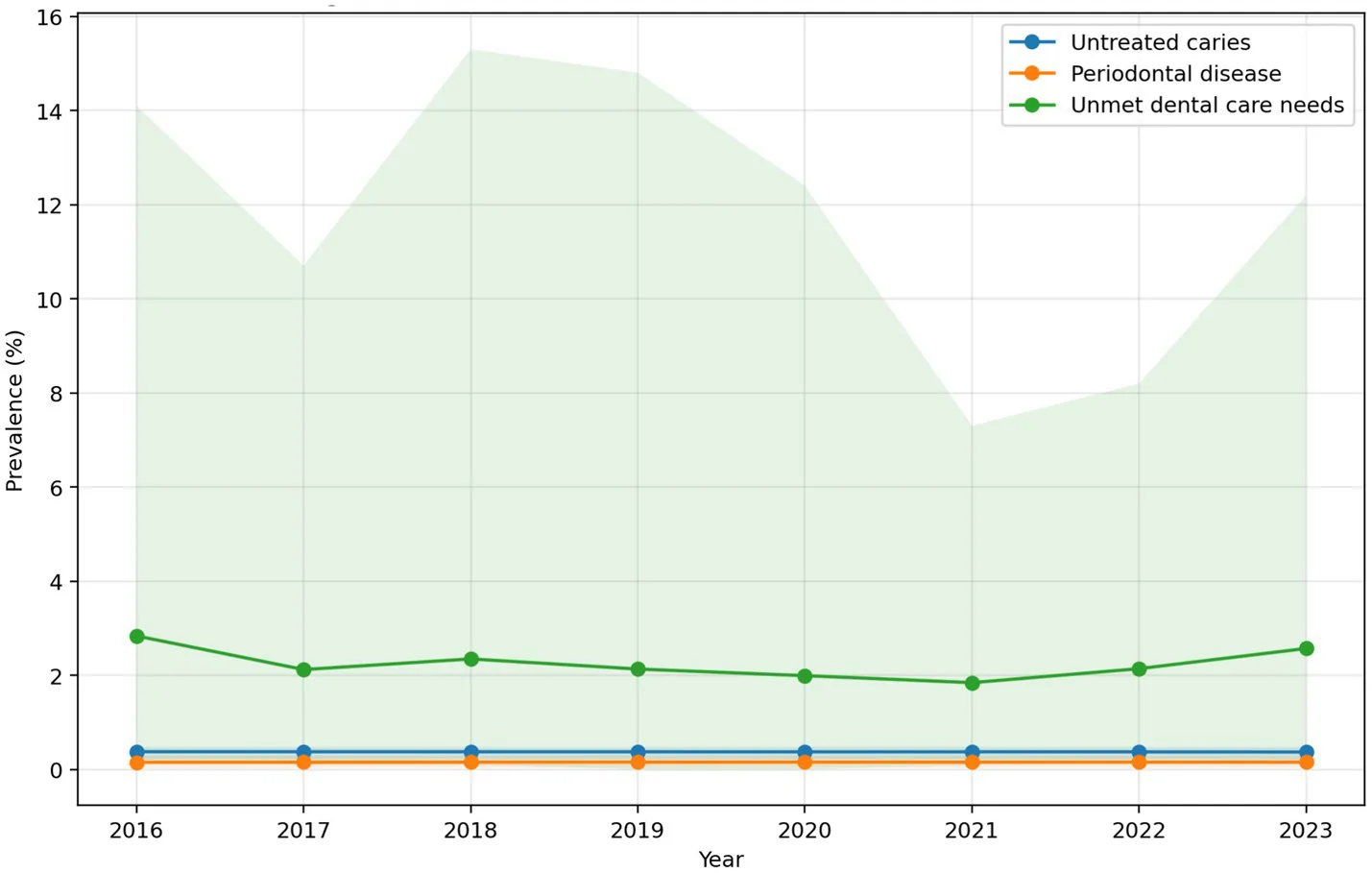
Evolution of oral health indicators in EU-27 during 2016–2023. Solid lines represent EU-27 averages for untreated dental caries, periodontal disease, and unmet dental care needs, while shaded areas indicate the minimum-maximum range across member states.

The prevalence of periodontal disease is much lower, and its value ranges approximately between 3.5 and 28.3%, with an average of 15.5%. This indicator also seems to have rather constant dynamics over time (Figure 3).

Unmet need for dental care services varies greatly depending on countries ranging from 0% up to 15.3%, with its average being approximately equal to 2.25%. It is worth noting that this indicator differs significantly from the previous two as it exhibits some changes related to time (Figure 3).

As can be observed, there are some noticeable disparities between countries. Caries cases not treated and oral care needs not met occur more often in Central and East European nations, whereas improved oral health outcomes appear more frequently in Western and Northern nations.

In general, such results illustrate considerable disparities in oral health indicators within EU member states, where improvements have been inadequate. This information is crucial to understand the econometric results provided below.

### 3.4 Panel regression results

The results for the fixed-effects panel regressions are provided in Table 3 based on the three dependent variables that were considered: untreated dental caries prevalence, periodontal disease prevalence, and unmet dental care needs.

**Table 3:** Results of the fixed-effects panel regression models examining the relationship between climate variables and oral health outcomes across EU-27 member states during the period 2016–2023.

Variable	Caries	Periodontal	Unmet
Temperature	0.087 (0.058)	0.068 (0.047)	−0.112 (0.186)
Extreme heat days	−0.004 (0.004)	0.001 (0.002)	0.003 (0.014)
Health expenditure	−0.106 (0.073)	0.094 (0.074)	−0.587* (0.315)
Observations	216	216	215
Within R-squared	0.056	0.032	0.044
Country fixed effects	Yes	Yes	Yes
Year fixed effects	Yes	Yes	Yes
Clustered SE by country	Yes	Yes	Yes

The dependent variables include untreated dental caries prevalence, periodontal disease prevalence, and unmet dental care needs. Coefficients are reported with standard errors in parentheses. *p < 0.10. All models include country and year fixed effects. Standard errors are clustered at the country level.

To help interpret the magnitude of each regression’s coefficient, a forest plot showing the regression coefficient and its 95% confidence interval is presented in Figure 4.

**Figure 4:**
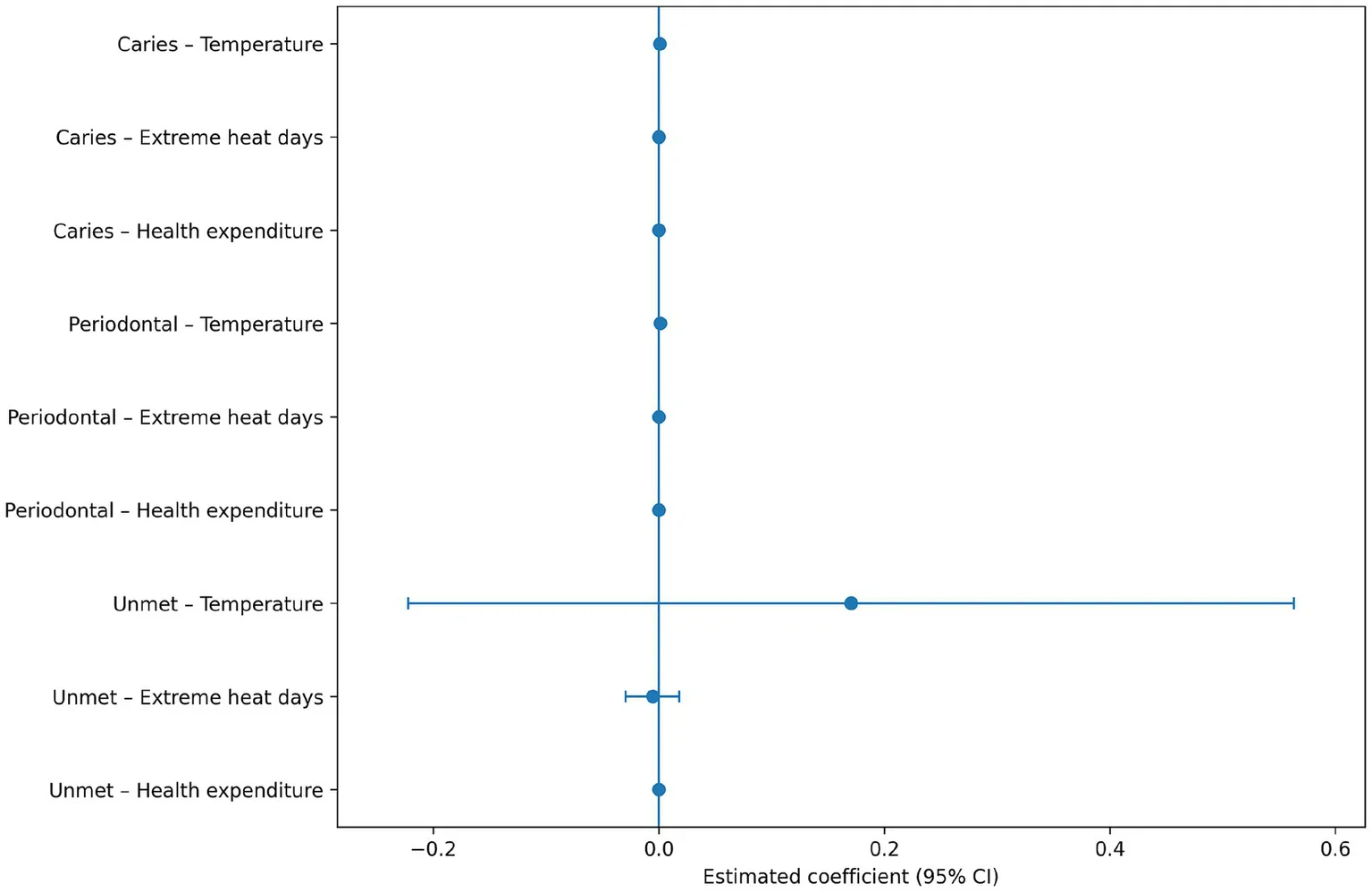
Forest plot of estimated coefficients from fixed-effects panel models. Points represent estimated coefficients, and horizontal lines indicate 95% confidence intervals. The figure summarizes the magnitude and direction of the associations between climate variables, public health expenditure, and oral health outcomes.

For untreated dental caries, temperature (β = 0.087, SE = 0.058; *p* = 0.142), extreme heat days (β = −0.004, SE = 0.004; *p* = 0.221), and expenditure (β = −0.106, SE = 0.073; *p* = 0.162) were not statistically significant. Wild-cluster bootstrap *p*-values were 0.127, 0.293, and 0.079, respectively.

For periodontal disease, temperature (β = 0.068, SE = 0.047; *p* = 0.162), extreme heat days (β = 0.001, SE = 0.002; *p* = 0.707), and expenditure (β = 0.094, SE = 0.074; *p* = 0.217) were not statistically significant. Wild-cluster bootstrap *p*-values were 0.113, 0.783, and 0.131.

For unmet dental care needs, temperature (β = −0.112, SE = 0.186; *p* = 0.554), extreme heat days (β = 0.003, SE = 0.014; *p* = 0.818), and expenditure (β = −0.587, SE = 0.315; *p* = 0.074) were not significant at 5%. The corresponding wild-cluster bootstrap *p*-values were 0.546, 0.805, and 0.125.

Overall, the reconstructed two-way fixed-effects analysis provided no evidence at the 5% level that within-country changes in mean annual temperature, extreme heat days, or general government health expenditure were associated with the three outcomes during 2016–2023.

The within R-squared values were 0.056, 0.032, and 0.044 for caries, periodontal disease, and unmet dental care, respectively. The low values are consistent with limited within-country outcome variation and indicate modest explanatory contribution of the time-varying covariates.

### 3.5 Sensitivity analyses

In order to check whether the primary specification of fixed effects is robust, a number of diagnostics was conducted together with alternative estimation of the models.

Quadratic specifications produced no significant squared-temperature terms (all *p* ≥ 0.199). Climate-by-expenditure interaction models likewise produced no interaction significant at 5% (all *p* ≥ 0.099). Therefore, the reconstructed data provide no evidence that the selected general expenditure indicator modified the short-term climate–oral-health association.

Small-cluster inference was assessed using 999-replication wild-cluster bootstrap *p*-values. None of the nine focal coefficients met the 5% threshold. This result supports the main interpretation while avoiding reliance solely on asymptotic cluster-robust standard errors with 27 countries.

Residual checks detected strong serial persistence in all outcomes and cross-sectional dependence in the periodontal model. These findings do not invalidate the fixed-effects estimand, but they limit precision and motivate cautious, exploratory interpretation.

Within-country variation was substantially smaller than between-country variation, particularly for the two modeled GBD prevalence outcomes. This feature helps explain the low within R-squared values and the wide uncertainty around the short-term exposure coefficients.

Accordingly, robustness checks do not support nonlinear or expenditure-modified climate associations. Multiple testing and the short time dimension remain important limitations (Table 4).

**Table 4:** Sensitivity analysis and diagnostic test results.

Test/Indicator	Caries	Periodontal	Unmet
Within R-squared	0.056	0.032	0.044
Wild p: temperature	0.127	0.113	0.546
Wild p: heat days	0.293	0.783	0.805
Wild p: expenditure	0.079	0.131	0.125
Residual AR(1)	0.649***	0.737***	0.422***
Pesaran CD *p*-value	0.052	<0.001	0.710
Quadratic temperature p	0.199	0.758	0.315
Temperature × expenditure p	0.099	0.517	0.271
Heat days × expenditure p	0.823	0.369	0.271

FE, fixed effects; RE = random effects; VIF, variance inflation factor. All models include country and year fixed effects. Standard errors are clustered at the country’s level. Wild *p*-values were calculated using 999 Rademacher replications clustered at the country level. AR(1) entries represent residual persistence coefficients; ***p < 0.001. Quadratic and interaction entries represent coefficient *p*-values. All specifications include country and year fixed effects.

### 3.6 Cross-sectional association between mean annual temperature and oral health outcomes

Complementary pooled analyses indicated geographical gradients between mean annual temperature and caries and periodontal prevalence. These estimates combine persistent differences between countries with temporal variation and therefore cannot identify a longitudinal climate effect. The patterns were not reproduced in the two-way fixed-effects models. Detailed pooled correlations, ordinary least-squares estimates, temperature-quartile summaries, and EU-27 annual averages were removed from the main text to maintain focus on the primary longitudinal analysis.

## 4 Discussion

In the two-way fixed-effects models, neither mean annual temperature nor extreme heat days were significantly associated with untreated caries of permanent teeth, periodontal diseases, or unmet dental-care needs. The principal finding is therefore the absence of detectable short-term within-country associations between the selected climate indicators and oral-health outcomes during 2016–2023. This result should not be interpreted as proof that climate-related exposures cannot affect oral health; rather, such effects were not identified at the national level over the available eight-year period.

The pooled analyses showed geographical gradients, but these patterns disappeared when persistent country characteristics and common year shocks were controlled. Pooled country-year associations can reflect stable differences in latitude, diet, socioeconomic conditions, health-service organization, preventive care, and other unmeasured characteristics. Their divergence from the fixed-effects estimates therefore reinforces the need to distinguish cross-country correlation from change occurring within countries.

General government health expenditure was not associated with any outcome at the 5% level. It is a broad health-financing measure rather than dental expenditure, periodontal prevention, oral-health coverage, or system capacity, and it may change because of either expenditure or GDP. The results therefore provide no evidence of a protective, causal, or climate-mitigating effect (26–33).

Biological pathways involving dehydration, salivary flow, oral homeostasis, and healthcare utilization remain plausible, but they operate primarily at the individual level and were not measured in this ecological analysis. National annual averages may mask short exposure windows, regional heat events, vulnerable subgroups, adaptive behaviors, and delayed disease processes.

The study is limited by its ecological design, model-based disease estimates, short temporal dimension, only 27 country clusters, centroid-based exposure assignment, residual serial dependence, and cross-sectional dependence in the periodontal model. The number of outcomes and predictors also raises a multiple-testing concern. Quadratic and interaction models were estimated but produced no associations at the 5% level; these sensitivity findings remain exploratory because each country contributes only eight annual observations.

Future research should use longer panels, regional or individual-level exposure data, oral-health-specific expenditure, service coverage, utilization, and biological measurements. Such studies are needed before policy conclusions can be drawn about climate adaptation or the role of healthcare financing in modifying climate-related oral-health risk.

## 5 Conclusion

This EU-27 panel study found no statistically significant association between short-term within-country variation in mean annual temperature or extreme heat days and untreated caries, periodontal diseases, or unmet dental-care needs during 2016–2023. The pooled geographical gradients were not reproduced in the longitudinal fixed-effects models and should not be interpreted as climate effects.

General government health expenditure was negatively associated with periodontal disease prevalence only. This outcome-specific observational result does not show that healthcare expenditure causes better oral health or mitigates climate-related risk, particularly because the indicator is not specific to dental services and one nominally significant coefficient was identified among several comparisons.

The short panel and ecological design limit statistical power and do not capture individual exposure, salivary mechanisms, delayed effects, or regional vulnerability. Longer-term, regional, and individual-level studies using oral-health-specific financing and service indicators are required before causal or policy conclusions can be supported.

## Data Availability

Publicly available datasets were analyzed in this study. This data can be found here: Eurostat. EU Statistics on Income and Living Conditions (EU-SILC), https://ec.europa.eu/eurostat/web/income-and-living-conditions; GBD 2019 Diseases and Injuries Collaborators, doi: 10.1016/S0140-6736(20)30925-9; and the ERA5 global reanalysis, doi: 10.1002/qj.3803.
