# Supplementary material for: Climate risk or healthcare investment? Explaining oral health outcomes across European Union member states
Source: Front Public Health. 2026 Sep 30;14:1939057. doi: 10.3389/fpubh.2026.1939057 (PMC13661443; doi:10.3389/fpubh.2026.1939057)
Supplement: Supplementary file 1 [file Supplementary_file_1.docx]

Supplementary Material

Climate Risk or Healthcare Investment? Explaining Oral Health Outcomes across European Union Member States

This supplement documents the reconstruction of the EU-27 country-year panel and reports the additional analyses requested during peer review. All outcomes are expressed on the 0-100 percentage-point scale.

# S1. Data reconstruction and definitions

The balanced panel covers 27 European Union member states and eight years (2016-2023; 216 country-year observations). Daily climate observations were downloaded for each country centroid and aggregated by calendar year. The analytical merge used country name/code and year. One Eurostat unmet-care observation was unavailable, giving N=215 for that outcome.

| Construct | Operational definition | Source |
| --- | --- | --- |
| Mean annual temperature | Annual mean of daily mean temperature at the country centroid | Copernicus Climate Data Store (ERA5) |
| Extreme heat days | Annual count of days with daily maximum temperature >30°C | Copernicus Climate Data Store (ERA5) |
| Untreated caries | Both sexes; all ages; caries of permanent teeth; prevalence | IHME GBD 2023 |
| Periodontal disease | Both sexes; age-standardized; periodontal diseases; prevalence | IHME GBD 2023 |
| Unmet dental care | Age 16+; unmet need due to cost, distance, or waiting list | Eurostat EU-SILC hlth_silc_09 |
| Health expenditure | Domestic general government health expenditure, % GDP | World Bank SH.XPD.GHED.GD.ZS |

# S2. Descriptive and within-country variation

| Variable | Mean | Overall SD | Within SD | Min | Max | N |
| --- | --- | --- | --- | --- | --- | --- |
| Mean annual temperature (°C) | 10.792 | 3.917 | 0.513 | 4.244 | 21.085 | 216 |
| Extreme heat days | 19.782 | 34.677 | 6.253 | 0.000 | 160.000 | 216 |
| Government health expenditure (% GDP) | 6.263 | 1.697 | 0.565 | 2.746 | 10.122 | 216 |
| Caries prevalence (%) | 37.480 | 6.870 | 0.302 | 22.077 | 47.658 | 216 |
| Periodontal prevalence (%) | 11.199 | 4.020 | 0.249 | 2.403 | 22.975 | 216 |
| Unmet dental care (%) | 3.502 | 3.216 | 1.178 | 0.100 | 14.200 | 215 |

Note: Within SD is calculated after removing each country mean and quantifies the temporal variation contributing to identification in the fixed-effects models.

# S3. Primary two-way fixed-effects models

All models include country and year fixed effects. Standard errors are clustered by country with the finite-sample correction G/(G-1) x (N-1)/(N-K), and coefficient p-values use a t distribution with 26 degrees of freedom. Wild p-values use 999 Rademacher replications clustered by country.

| Outcome | Predictor | Coefficient | Clustered SE | p-value | Wild p-value |
| --- | --- | --- | --- | --- | --- |
| Caries | Temperature | 0.087 | 0.058 | 0.142 | 0.127 |
| Caries | Extreme heat days | -0.004 | 0.004 | 0.221 | 0.293 |
| Caries | Health expenditure | -0.106 | 0.073 | 0.162 | 0.079 |
| Periodontal | Temperature | 0.068 | 0.047 | 0.162 | 0.113 |
| Periodontal | Extreme heat days | 0.001 | 0.002 | 0.707 | 0.783 |
| Periodontal | Health expenditure | 0.094 | 0.074 | 0.217 | 0.131 |
| Unmet dental care | Temperature | -0.112 | 0.186 | 0.554 | 0.546 |
| Unmet dental care | Extreme heat days | 0.003 | 0.014 | 0.818 | 0.805 |
| Unmet dental care | Health expenditure | -0.587 | 0.315 | 0.074 | 0.125 |

# S4. Model fit and residual diagnostics

| Outcome | N | Clusters | Within R² | Residual AR(1) | AR p | Pesaran CD | CD p |
| --- | --- | --- | --- | --- | --- | --- | --- |
| Caries | 216 | 27 | 0.056 | 0.649 | <0.001 | -1.95 | 0.052 |
| Periodontal | 216 | 27 | 0.032 | 0.737 | <0.001 | 10.07 | <0.001 |
| Unmet dental care | 215 | 27 | 0.044 | 0.422 | <0.001 | -0.37 | 0.710 |

Residual serial persistence was detected for all three outcomes. Cross-sectional dependence was detected for periodontal disease. These diagnostics motivate the cautious interpretation and small-cluster robustness analyses reported in the manuscript.

# S5. Nonlinear temperature sensitivity analysis

| Outcome | Term | Coefficient | Clustered SE | p-value |
| --- | --- | --- | --- | --- |
| Caries | Temperature | -0.0515 | 0.1143 | 0.656 |
| Caries | Temperature² | 0.0069 | 0.0052 | 0.199 |
| Periodontal | Temperature | 0.0799 | 0.0513 | 0.131 |
| Periodontal | Temperature² | -0.0006 | 0.0020 | 0.758 |
| Unmet dental care | Temperature | 0.5228 | 0.6635 | 0.438 |
| Unmet dental care | Temperature² | -0.0317 | 0.0309 | 0.315 |

No squared-temperature coefficient was statistically significant at the 5% level.

# S6. Climate-by-expenditure interaction analysis

| Outcome | Interaction term | Coefficient | Clustered SE | p-value |
| --- | --- | --- | --- | --- |
| Caries | Temperature × expenditure | 0.0225 | 0.0131 | 0.099 |
| Caries | Heat days × expenditure | -0.0003 | 0.0011 | 0.823 |
| Periodontal | Temperature × expenditure | 0.0114 | 0.0173 | 0.517 |
| Periodontal | Heat days × expenditure | -0.0017 | 0.0018 | 0.368 |
| Unmet dental care | Temperature × expenditure | 0.0929 | 0.0826 | 0.271 |
| Unmet dental care | Heat days × expenditure | -0.0068 | 0.0060 | 0.271 |

No climate-by-expenditure interaction was statistically significant at the 5% level. Accordingly, the analysis provides no evidence that the broad government expenditure indicator modified the short-term climate association.

# S7. Multiple testing and interpretation

The three primary outcomes and three focal predictors yield nine principal coefficient tests, in addition to exploratory nonlinear and interaction terms. None of the nine primary coefficients met the 5% threshold under wild-cluster bootstrap inference. Interpretation therefore emphasizes the overall null pattern rather than isolated nominal p-values. The sensitivity analyses are hypothesis-generating.

# S8. Reproducibility note

Country-centroid coordinates, daily-to-annual aggregation rules, indicator identifiers, outcome scaling, model specification, finite-sample covariance correction, and bootstrap settings are reported above to permit independent reconstruction. The centroid exposure is a national proxy and does not represent population-weighted or within-country regional exposure.

# S9. Exploratory pooled correlations and OLS regressions

These analyses were moved from the main manuscript because they combine persistent between-country differences with annual within-country variation. They are descriptive and must not be interpreted as estimates of longitudinal or causal climate effects.

| Outcome | N | r | p-value | OLS slope | 95% CI | Intercept | R² |
| --- | --- | --- | --- | --- | --- | --- | --- |
| Caries prevalence | 216 | 0.265 | <0.001 | 0.465 | [0.239, 0.692] | 32.459 | 0.070 |
| Periodontal prevalence | 216 | -0.330 | <0.001 | -0.339 | [-0.468, -0.209] | 14.852 | 0.109 |
| Unmet dental care | 215 | 0.028 | 0.680 | 0.023 | [-0.087, 0.134] | 3.252 | 0.001 |

Figure S1. Exploratory pooled relationship between mean annual temperature and untreated caries prevalence.

Figure S2. Exploratory pooled relationship between mean annual temperature and periodontal disease prevalence.

# S10. Temperature-quartile analysis

| Quartile | N | Temp min | Temp max | Mean temp | Mean caries | Mean periodontal | Mean unmet |
| --- | --- | --- | --- | --- | --- | --- | --- |
| Q1 | 54 | 4.244 | 8.170 | 6.691 | 34.995 | 11.942 | 3.959 |
| Q2 | 54 | 8.188 | 9.972 | 9.101 | 37.628 | 12.961 | 2.969 |
| Q3 | 54 | 9.976 | 12.048 | 11.001 | 36.731 | 11.424 | 2.480 |
| Q4 | 54 | 12.059 | 21.085 | 16.374 | 40.568 | 8.468 | 4.621 |

Quartiles contain 54 country-year observations each. The gradients are pooled descriptive patterns and do not isolate change within countries.

# S11. EU-27 annual averages

| Year | Temperature | Heat days | Expenditure | Caries | Periodontal | Unmet |
| --- | --- | --- | --- | --- | --- | --- |
| 2016 | 10.467 | 15.926 | 5.901 | 37.728 | 11.169 | 4.496 |
| 2017 | 10.371 | 19.963 | 5.824 | 37.644 | 11.213 | 3.670 |
| 2018 | 10.957 | 18.556 | 5.867 | 37.564 | 11.258 | 3.644 |
| 2019 | 10.963 | 20.667 | 5.964 | 37.514 | 11.289 | 3.326 |
| 2020 | 11.154 | 18.852 | 6.779 | 37.428 | 11.264 | 3.008 |
| 2021 | 10.209 | 20.556 | 6.935 | 37.515 | 11.182 | 2.937 |
| 2022 | 11.023 | 23.296 | 6.476 | 37.363 | 11.141 | 3.341 |
| 2023 | 11.188 | 20.444 | 6.360 | 37.086 | 11.074 | 3.574 |

Figure S3. Annual EU-27 means for reconstructed temperature and extreme heat-day indicators, 2016-2023.
